# Effects of enzymatic hydrolysis on physicochemical property and antioxidant activity of mulberry (*Morus atropurpurea* Roxb.) leaf protein

**DOI:** 10.1002/fsn3.2474

**Published:** 2021-08-25

**Authors:** Chongzhen Sun, Yangwei Shan, Xin Tang, Duo Han, Xiyang Wu, Hui Wu, Marzieh Hosseininezhad

**Affiliations:** ^1^ Department of Food Science and Engineering Jinan University Guangzhou China; ^2^ College of Food Science and Engineering South China University of Technology Guangzhou China; ^3^ Department of Food Biotechnology Research Institute of Food Science and Technology (RIFST) Mashhad Iran

**Keywords:** antioxidant activity, enzymatic hydrolysis, FTIR analysis, molecular weight, Mulberry leaf protein

## Abstract

To improve the antioxidant efficiency of mulberry leaf protein (MLP), alcalase, protamex, papain, flavourzyme, neutrase, and trypsin were used to hydrolyze MLP. The yield of soluble peptides, secondary structures, molecular weight distributions, and antioxidant activities of MLP hydrolysates (MLPHs) were investigated. Results showed that the native MLP was rich in the fraction above 6.5 kDa and was mainly composed of β‐sheets, while MLPHs were abundant in the fractions of 0.3–0.6 kDa and 0.6–6.5 kDa and were mainly composed of disordered coils and β‐folds. Limited hydrolysis of MLP could lead to better antioxidant activity than extensive hydrolysis. After enzymatic hydrolysis, the content of total sugar and total phenol in MLP increased significantly. MLP hydrolysates prepared with neutrase, alcalase, and protamex were preferable to other enzymes. Meanwhile, an enzyme to substrate level of 1% and a hydrolysis time of 2 hr were the optimum conditions to obtain higher antioxidant hydrolysates using neutrase.

## Graphical abstract

Six different enzymes were used to hydrolyze mulberry leaf protein. The yield of soluble peptides, secondary structures, molecular weight distributions, total sugar and phenol, and antioxidant activity of different hydrolysates (MLPH) were analyzed. MLPH prepared with neutrase, alcalase, and protamex were preferable to other enzymes, and neutrase hydrolysates showed the best antioxidant activity.

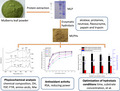

## 1 INTRODUCTION

Free radicals are highly reactive species that can induce fatty acid and lipid oxidation in food. This oxidation not only leads to the deterioration of quality attributes, such as flavor, aroma, texture, and color but also to loss of nutritive value and food spoilage. (Elias et al., 2008; Kou et al., 2013) In addition, free radical reactions can lead to the formation of reactive oxygen species (ROS), which play critical roles in various human diseases. (Dedon & Tannenbaum, 2004; Fubini & Hubbard, 2003; Yan et al., 2015) Therefore, antioxidant additives that can significantly inhibit oxidative reactions and improve the nutritional value of sensitive food products are important in the food industry. In recent years, natural antioxidants, such as proteins and protein hydrolysates have drawn the attention of researchers due to their wide distribution, excellent activity, and low toxicity. (Pownall et al., 2010) In addition, protein hydrolysates are considered suitable protein sources for human nutrition, as hydrolysates contain many small peptides, which can be absorbed more effectively than either intact protein or free amino acids. (Jin et al., 2015; Manninen, 2009) Moreover, it is widely recognized that antioxidant peptides and hydrolysates derived from proteins have low allergenicity and high activity. (Elias et al., 2008; Yan et al., 2015) Bioactive peptides and hydrolysates can be generated by in vitro enzymatic hydrolysis, which, compared with other methods, is a milder process with fewer undesirable side effects, improved extraction yield, and more bioactive components. (Feng et al., 2018; He et al., 2015; Sila & Bougatef, 2016) Protein hydrolysates with strong antioxidant activities have been produced from various plant sources, such as flaxseed, (Karamać et al., 2016) cherry seed, (García et al., 2015) alfalfa leaf, (Xie et al., 2008) *Dendrobium aphyllum*, (Liu et al., 2018) and rice bran, (Thamnarathip et al., 2016) using exogenous proteases. Several studies have demonstrated that the specificity of the enzyme and the conditions of hydrolysis (pH, enzyme to substrate ratio, and temperature) greatly influence the molecular weights (Mw), secondary structures, and amino acid compositions and sequences of bioactive peptides, and thus their biological activities. (He et al., 2015; Jin et al., 2015; Sila & Bougatef, 2016) Therefore, it is very important to choose a suitable enzyme and determine the optimal conditions for bioactive protein hydrolysates preparation.

Mulberry leaves rich in protein and fiber and have been used for a long time as functional foods. (Sánchez‐Salcedo et al., 2017) Traditionally, mulberry leaves have been used to feed silkworms and herbivorous animals because of their high nutrition and palatability. (Andallu et al., 2014; Jeszka‐Skowron et al., 2014) In some Asian countries, particularly Japan, Korea, India, and Thailand, mulberry leaves are not only considered as potential plant protein sources for animal production, (Kandylis et al., 2009; Vu et al., 2011) but are also used as herbal medicines and have been consumed as infusion and herbal tea to treat fevers, protect the liver and improve eyesight. (He et al., 2018; Phoonan et al., 2014; Wanyo et al., 2011) Nowadays, mulberry leaves have been consumed as functional foods or food nutritional additives to produce mulberry leaf noodles, soup, bean curd, wine, beverage, puffed snacks, and tablets, etc. (Phoonan et al., 2014; Sánchez‐Salcedo et al., 2017; Thirugnanasambandham et al., 2015; Wanyo et al., 2011; Yoshihashi et al., 2010) Considering the wide availability and edibility, increasing interests have been focused on mulberry leaves. Until now, several functional components of mulberry leaf, including alkaloids, phenols, and flavonoids with hypoglycemic, anti‐inflammatory and antioxidant activities have been studied in detail. (Chang et al., 2016; Hao et al., 2018; Sanchez‐Salcedo et al., 2015; Zhang et al., 2016) On the other hand, mulberry leaf is a good source of protein, which accounts for 17%–25% of dry matter. (Sun et al., 2015, 2021; Zhang et al., 2014) Previously, we have focused on the extraction of mulberry leaf protein (MLP) as well as on the determination of its physicochemical properties and antioxidant activities. (Sun et al., 2015, 2017) Results suggested that MLP had excellent antioxidant activities, including DPPH and ABTS radical scavenging activity (RSA), chelating ability, and reducing power. However, there is little information about the antioxidant properties of MLP hydrolysates (MLPHs). (Kou et al., 2013).

Therefore, the objectives of this work were (1) to prepare MLPHs with different proteases (including alcalase, protamex, papain, flavourzyme, neutrase, and trypsin), (2) to investigate the effects of enzymatic hydrolysis on the physicochemical properties (including secondary structure, amino acid composition, and molecular weight) and antioxidant activities (including radical scavenging ability and reducing power) of MLPHs, (3) to evaluate the relationship between the physicochemical properties and antioxidant activity, and (4) to select the most appropriate enzyme for the preparation of antioxidant peptides from MLP.

## 2 MATERIALS AND METHODS

### 2.1 Sample and Regents

Mulberry (*Morus atropurpurea* Roxb.) leaves were supplied by Guangdong Academy of Agricultural Sciences Institute of Sericulture and Agricultural Products Processing. Alcalase 3.0T (3 AU g^‐1^), Flavourzyme (500 LAPU g^‐1^), Protamex (1.5 AU g^‐1^), and Neutrase (0.8 AU g^‐1^) were purchased from Novo Enzyme Co. (Denmark). Trypsin (250 U mg^‐1^) and papain (800 U mg^‐1^) were obtained from Biotopped Co. (Beijing, China). L‐glutathione (GSH), 1,1‐diphenyl‐2‐picrylhydrazyl (DPPH), 2,2'‐azino‐bis(3‐ethylbenzothiazoline‐6‐sulfonic acid) diammonium salt (ABTS), and ferrozine were purchased from Sigma (St. Louis, MO., USA). Other reagents of analytical grade were obtained from Aladdin Co. (Shanghai, China).

### 2.2 Extraction of MLP

Mulberry leaf protein was prepared by the albumin extraction method described in our previous work. (Sun et al., 2017) The protein content of MLP, as determined by the micro Kjeldahl method was 75.20 ± 1.24%. To remove the polyphenols and improve the purity, MLP was further purified by macroporous adsorption resin DA201‐C analysis using the method of Wu et al. (Wu et al., 2016) Namely, 50 ml of albumin solution at a concentration of 5 mg/ml was absorbed by macroporous adsorption resin DA 201‐C (Jiangyin Organic Chemical Plant, Jiangyin, China), and eluted by 75% ethanol solution. The eluent was further evaporated by rotary evaporator at 50°C with a speed of 60 rpm and freeze‐dried, then purified MLP was obtained.

### 2.3 Enzymatic Hydrolysis of MLP

The purified MLP was dissolved in distilled water (1%, *w/v*), and the dispersion was hydrolyzed using six kinds of proteases at the same enzyme to substrate ratio of 60,000 U/g). To inactivate protease inhibitors contained in the MLP, the slurry was heated at 95°C for 10 min prior to hydrolysis. The pH was adjusted to the optimal level for each enzyme with 0.5 M NaOH prior to protease addition. The optimal hydrolysis conditions were: Alcalase, 55°C, pH 8.0; Protamex and papain, 55°C, pH 7.0; Flavourzyme, 50°C, pH 7.0; Neutrase, 45°C, pH 7.0; trypsin, 37°C, pH 8.0. The pH was readjusted every 5 min from 0 to 25 min, and optimized every 20 min from 25 to 245 min. Reactions were terminated by heating at 95°C for 10 min. When the temperature dropped to 25°C, the pH value was adjusted to 7.0. Then the mixture was centrifuged (8,000 *g*, 10 min) and the supernatant was dialyzed using dialysis bag (Molecular weight cutoff was 100 Da), and lyophilized to produce MLPHs, which were stored at −20°C.

### 2.4 Analysis of Chemical Compositions

Protein content of MLP and MLPHs was determined by the Kjeldahl methods, (Association of Official Analytical Chemists, 1989) the protein/nitrogen coefficient used was 6.25. Total sugar content was determined by phenol–sulfuric acid method. (Cuesta et al., 2003) Total phenolic content (TPC) was measured by the Folin‐phenol method, (Ma et al., 2014) the results were expressed as grams of gallic acid equivalents (GAE) per hundred grams of dry material (g GAE/100g). Protein yield was described as follows:

$$\text{protein}\;\text{yield}\;\left(\%\right)=\frac{\text{weight}\;\text{of}\;\text{protein}\;\text{material}\times \text{protein}\;\text{content}}{\text{weight}\;\text{of}\;\text{material}\times \text{protein}\;\text{content}\;\text{of}\;\text{material}}\times 100\%$$

### 2.5 Determination of the Degree of Hydrolysis (DH)

Since all the reaction pH values were equal to or above 7.0, the degree of hydrolysis (DH) was measured by the pH‐stat method described by Wu et al. (Wu et al., 2016) The total amount of peptide bonds in the protein substrate is 8.16 mmol/g.

### 2.6 Analysis of the Yield of Soluble Peptide (YSP)

The concentration of soluble peptides in MLPHs was determined by the method of Wu et al. (Wu et al., 2016) Reduced GSH (0.0–2.0 g/L) was used to establish a standard curve. The soluble peptide concentration (*T_s_*) was calculated from the reduced GSH standard curve. The yield of soluble peptides (%) was calculated as (*T_s_*/*T_p_*) × 100, where *T_p_* represents the protein concentration of the substrate.

### 2.7 Fourier Transform infrared Spectroscopy (FTIR) Analysis

The FTIR spectra of the MLPHs were recorded at 25°C using a VERTEX 70 FTIR Spectrometer (Bruker, Germany) with a resolution of 2 cm^‐1^. All samples were analyzed in the solid state. To study the amide I region (1600–1700 cm^‐1^) of samples, the second‐order derivative spectrum was performed using Origin Pro 8.6.

### 2.8 Molecular weight determination

Molecular weights (Mw) were determined by gel permeation chromatography method described in our previous work. (Sun et al., 2018) A calibration curve was made from the log Mw of the markers and their respective elution times (*y* = −3.472*x* + 30.005, R^2^ = 0.9917). The relative content of each fraction was expressed as the percentage area of its chromatogram peak.

### 2.9 Amino acid analysis

Amino acid analysis of MLPHs was performed on an A300 auto amino acid analyzer using the method described in our previous work. (Sun et al., 2018) The results of amino acid composition analysis were expressed as g/kg sample.

### 2.10 Determination of antioxidant activity

The radical scavenging activity (RSA) of samples was investigated using DPPH, ABTS, and superoxide radical scavenging assays. DPPH radical scavenging activity was determined according to Chen et al. (Chen et al., 2013). Superoxide radical scavenging assay was measured using the method of Bamdad, et al., (Bamdad & Chen, 2013) ABTS^+^. scavenging activity was measured according to Garcia et al. (García et al., 2015) The ferric ion reducing power of MLP and MLPHs at 0.5 g/L was investigated according to Xie et al. (Xie et al., 2008) All experiments were carried out in triplicate. The sample concentration ranged from 0.05 to 1.0 mg/ml.

### 2.11 Optimization of enzymatic hydrolysis conditions

After analyzing the antioxidant activity of single enzymatic hydrolysates, the protease with better performance was selected to analyze the antioxidant activity under different hydrolysis time, and the enzyme with higher activity was selected to further optimize its enzymatic hydrolysis conditions. The effects of the amount of protease (1%, 2%, 3%, 4%) and substrate concentration (10, 20, 30, 40 mg/ml) on antioxidant activity were investigated.

### 2.12 Statistical analysis

The data are presented as the mean ± standard deviation (*SD*). Statistical calculations were performed by one‐way analysis of variance using SPSS Statistics V17.0. Duncan's multiple range tests were used to identify significant differences (*p* < .05) among treatment means. A two‐tailed Pearson's correlation test was conducted to determine correlations.

## 3 RESULTS AND DISCUSSION

### 3.1 DH and YSP analysis of MLPHs

**FIGURE 1:**
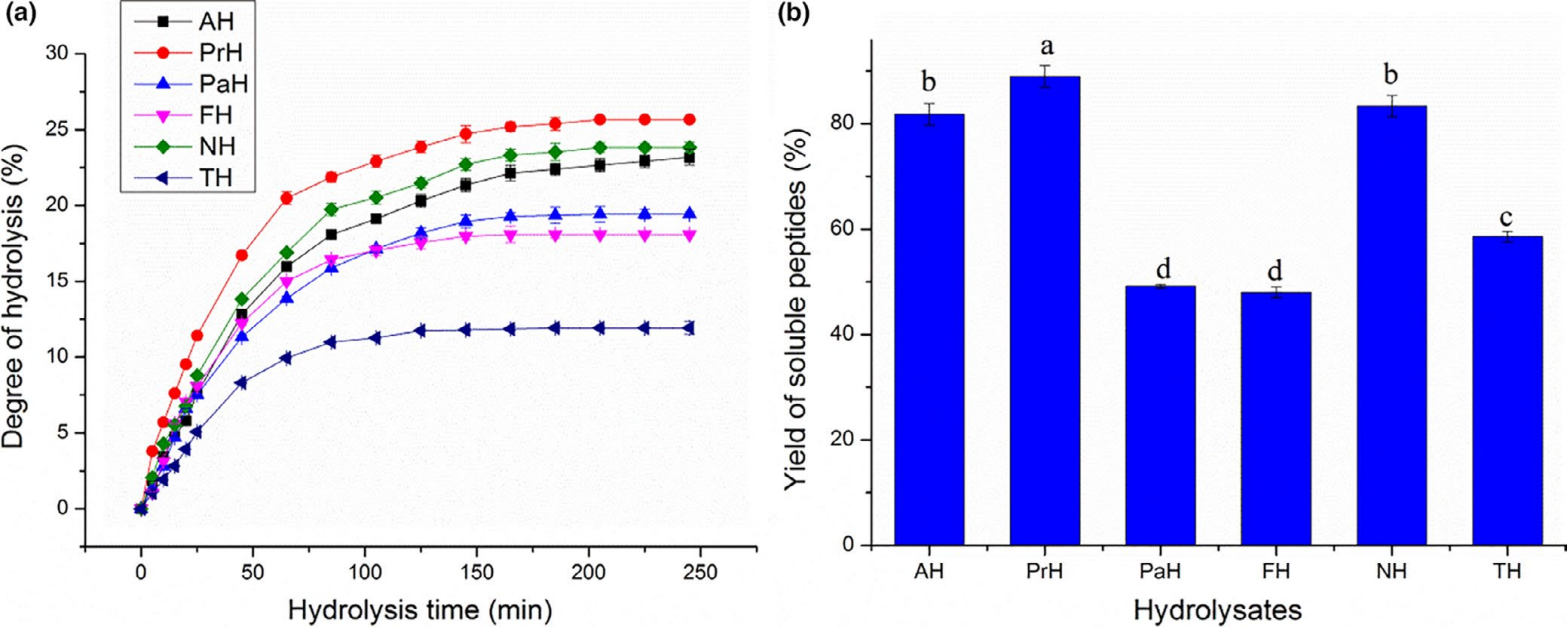
Degree of hydrolysis (a) and soluble peptide yield (b) of MLP with different individual proteases. MLP: mulberry leaf protein; AH, PrH, PaH, FH, NH and TH represent the hydrolysates of MLP hydrolyzed by alcalase, protamex, papain, flavourzyme, neutrase and trypsin, respectively. Different letters denote significant differences (*p* < .05)

Enzymatic hydrolysis affects peptide bioactivity by DH and enzymes. (Li et al., 2014) The DH affects the size and the amino acid composition of peptides, which could modulate their biological activities. (Sila & Bougatef, 2016) The changes in DH of MLPH after various incubation times are shown in Figure 1a. The enzyme type and hydrolysis time were the major factors affecting the degree of hydrolysis. (Thamnarathip et al., 2016) As shown in Figure 1a, trypsin hydrolysis proceeded at a low rate, with DH reaching only 11.93% after 245 min of incubation. The hydrolysis rate with the other proteases increased in a linear fashion (R^2^=0.967 ~ 0.996) during the first 45 min of incubation, increased at a slower rate from 65 to 145 min, and then plateaued. During the initial hydrolysis, peptide bonds were quickly fractured and a large amount of amino nitrogen was produced. Therefore, the DH increased rapidly. As the hydrolysis continued, the peptide bonds were ruptured at a slower rate and the DH became more stable. The low DH value of trypsin hydrolysate (TH) could be attributed to its specificity in the cleavage of peptide bonds. Among the six hydrolysates, protamex hydrolysate (PrH) produced the highest DH value, followed by neutrase hydrolysate (NH), and alcalase hydrolysate (AH). Protamex, neutrase, and alcalase are all endoproteases that have been widely used to hydrolyze proteins into bioactive peptides. (Bamdad et al., 2011; Ren et al., 2014) The efficient hydrolysis observed in PrH, NH, and AH demonstrates their high proteolytic capacities toward MLP. As shown in Figure 1, the DH and YSP values for PrH, AH, and NH were higher than those of papain hydrolysate (PaH), flavourzyme hydrolysate (FH), and TH. PaH and FH showed significantly higher DH values, but significantly lower YSP values than TH (*p* < .05). As a result, and contrary to the results of Wu et al., (Wu et al., 2016) there was no significant positive correlation between DH and YSP (r = .528, Table S1).

### 3.2 Chemical composition of MLP and MLPHs

The chemical composition of MLP and MLPHs is shown in Table 1. All the hydrolysates had protein contents above 64% with TH being the lowest at 64.79%, while AH, PrH, and NH had the highest at 80.39%, 79.55%, and 79.52%, respectively. These values were lower than those obtained from wheat germ globulin hydrolysates using alcalase after extended prolonged period of hydrolysis, (Wu et al., 2016) but higher than that recorded for 4 hr pepsin hydrolysates of canola meal protein. (Alashi et al., 2014) The protein yield can also be used as an indicator of the efficiency of enzymatic hydrolysis. (Jin et al., 2015) As shown in Table 1, the protein yield of MLPHs is higher than 50%, indicating that most proteins can be hydrolyzed by enzymes and converted into peptides. In addition, there was a significant positive correlation between protein content and protein yield (*r* = .999, Table S1), which was consistent with the results of Thamnarathip et al. (Thamnarathip et al., 2016) After enzymatic hydrolysis, the content of total sugar and total phenol in MLP increased significantly. This is mainly due to the degradation of polyphenols and polysaccharides during the enzymatic hydrolysis process, resulting in the formation of small monosaccharides and some free phenolic acids. At the same time, in the native MLP, some phenols can combine with the protein in covalent or non‐covalent forms. During the enzymatic hydrolysis, more phenols are released and become free phenols. (Thamnarathip et al., 2016).

**TABLE 1:** The chemical composition and degree of hydrolysis of mulberry leaf protein hydrolysates

Enzymes	Samples^1^	DH (%)^3^	Protein content (%)	Protein yield (%)	Total sugar (g/100 g)	TPC^2^ (g GAE/100 g)
‐‐	MLP	‐‐	79.20 ± 3.12^a^	‐‐	4.04 ± 0.31^d^	2.09 ± 0.05^c^
Alcalase	AH	23.19 ± 1.06^a^	80.39 ± 1.31^a^	93.06 ± 2.12^a^	5.19 ± 0.16^c^	3.54 ± 0.02b^a^
Protamex	PrH	25.67 ± 0.60^a^	79.55 ± 2.63^a^	92.91 ± 2.06^a^	6.42 ± 0.40^b^	3.58 ± 0.19^a^
Papain	PaH	19.44 ± 0.02^b^	70.78 ± 3.00^b^	66.43 ± 1.21^c^	6.12 ± 0.62^b^	2.64 ± 0.01^b^
Flavourzyme	FH	18.08 ± 0.19^b^	74.73 ± 2.69^b^	77.47 ± 1.56^b^	5.08 ± 0.39^c^	2.68 ± 0.11^b^
Neutrase	NH	23.83 ± 0.22^a^	79.52 ± 3.21^a^	91.57 ± 3.06^a^	6.42 ± 0.28^b^	3.33 ± 0.24^a^
Trypsin	TH	11.93 ± 0.42^c^	64.79 ± 2.31^c^	50.62 ± 2.02^d^	8.03 ± 0.47^a^	3.83 ± 0.12^a^

Note

Data are expressed as mean ± *SD*, the superscripts following each figure in the same column indicate significant differences at *p* < .05, *n* = 3. The superscripts (a,b, c and d) following each data in the same column indicate significant differences at *p* < .05

^1^ MLP: mulberry leaf protein; AH, PrH, PaH, FH, NH, TH: the hydrolysates of MLP hydrolyzed by alcalase, protamex, papain, flavourzyme, neutrase and trypsin, respectively.

^2^ TPC: total phenolic content, grams of gallic acid equivalents (GAE) per 100 grams of dry material (g GAE/100g).

^3^ DH: the maximum degree of hydrolysis at 4 hr; ND means not detected.

### 3.3 Molecular weight distributions

**FIGURE 2:**
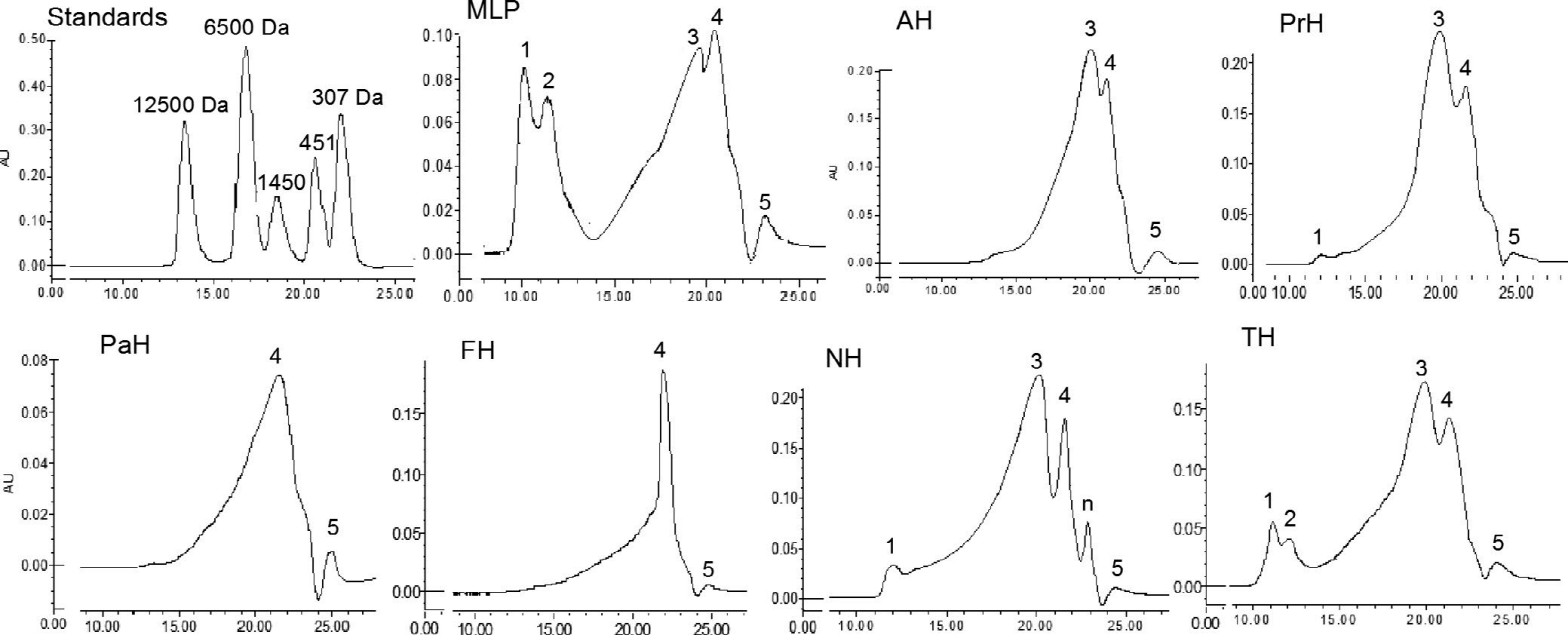
High performance gel permeation chromatography of mulberry leaf protein hydrolysates. MLP: mulberry leaf protein; AH, PrH, PaH, FH, NH, TH: the hydrolysates of MLP hydrolyzed by alcalase, protamex, papain, flavourzyme, neutrase and trypsin, respectively; 1–5 is corresponding to the molecular weight range of Table 2

The Mw distributions of MLP and MLPHs are shown in Table 2 and Figure 2. MLP was divided into five fractions. After hydrolysis, the percentage of fractions 1 (>35 kDa) and 2 (6.5–35 kDa) dramatically decreased, while fractions 3 (0.5–6.5 kDa) and 4 (0.3–0.5 kDa) significantly increased, and the contents of fraction 5 (<0.3 kDa) increased slightly. The results confirmed that enzymatic treatment changed the larger protein into smaller polypeptides, oligopeptides, and free amino acids. Only TH retained a portion of MLP in fractions 1 and 2 (14.90%), indicating that trypsin hydrolysis could not completely destroy the protein structure of MLP. Fraction 2 of MLP was thoroughly degraded by the other five enzymes, and fractions 1 and 3 was completely digested by papain and Flavourzyme (Figure 2). AH, PrH, and NH displayed similar Mw distributions, and were abundant in polypeptides (0.5–6.5 kDa). The chromatograms of AH and PrH were similar, while NH presented a new peak (*n*) between 4 and 5 components (Figure 2). PaH and FH are rich in oligopeptides (0.3–0.5 kDa)while PaH and FH were rich in oligopeptides (0.3–0.6 kDa). These results indicated that different enzymes exerted varied effects on MLP degradation.

**TABLE 2:** The molecular weight distribution and IC50 value of MLP and MLPHs

Samples	1	2	3	4	5	IC50 value of radical scavenging activity (mg/ml)		
	(>35 kDa)	(6.5–35 kDa)	(0.5–6.5 kDa)	(0.3–0.5 kDa)	(<0.3 kDa)	DPPH.	O_2_^‐^	ABTS^+^
MLP	25.27	20.82	36.50	15.11	2.3	0.181	0.68	1.286
AH	1.09	ND	58.93	31.16	8.82	0.092	0.378	0.876
PrH	1.56	ND	56.08	32.07	9.29	0.091	0.411	0.844
PaH	ND	ND	ND	84.50	15.50	0.307	0.842	2.377
FH	ND	ND	ND	94.61	5.35	0.252	0.653	1.676
NH	1.74	ND	59.08	26.88	12.30	0.087	0.351	0.816
TH	9.27	5.66	55.02	24.32	5.73	0.099	0.468	1.144

Abbreviations: MLP, mulberry leaf protein; MLPHs, hydrolysates of MLP; IC50, inhibitory concentration of 50%; AH, PrH, PaH, FH, NH, TH, the hydrolysates of MLP hydrolyzed by alcalase, protamex, papain, flavourzyme, neutrase and trypsin, respectively.

### 3.4 FTIR analysis

**FIGURE 3:**
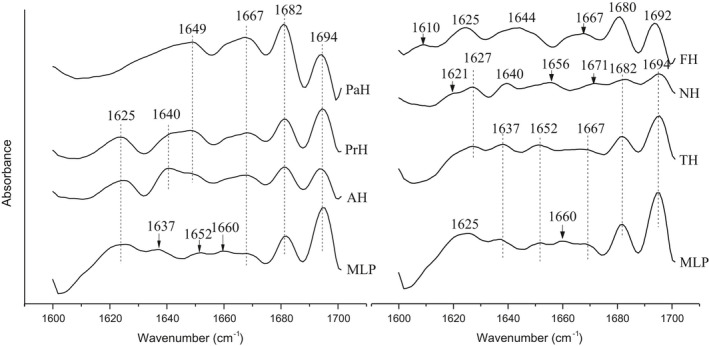
The second‐derivative FTIR spectra in the amide I region of MLP and its hydrolysates. MLP: mulberry leaf protein; AH, PrH, PaH, FH, NH, and TH: the hydrolysates of MLP hydrolyzed by alcalase, protamex, papain, flavourzyme, neutrase and trypsin, respectively

FTIR detects relative changes in the protein secondary structure by analyzing the amide I region (1600–1700 cm^‐1^). (Murariu et al., 2009) The second‐derivative FTIR spectra of MLP and MLPHs are shown in Figure 3. MLP was characterized by 7 bands, which were assigned to β‐sheets (1694, 1667, 1637, and 1625 cm^‐1^), a parallel β‐fold (1682 cm^‐1^), a β‐turn (1,660 cm^‐1^), and unordered structures and α‐helices (1652 cm^‐1^). (Bamdad et al., 2011; Uluko et al., 2015) β‐sheets are the major secondary structures of MLP. We note that the 1,660 cm^‐1^ band for MLP, which we assigned to carbonyl stretching of the glutamine side chain, disappeared after hydrolysis, meaning that glutamine residues were gradually exposed and converted to glutamic acid by deamidaion. (Bamdad & Chen, 2013; Bamdad et al., 2011) AH and PrH were also rich in β‐sheets. In addition to the same peaks as MLP at 1625, 1667, 1682, and 1694 cm^‐1^, the absorption bands at 1637 and 1652 cm^‐1^ disappeared and were replaced by two new bands at 1,640 and 1649 cm^‐1^ (unordered coils). Moreover, the absorption intensities at 1625 and 1667 cm^‐1^ (β‐sheets) became more distinct. The spectrum of FH was remarkably different from MLP, with three new structures, including a side chain vibration (1,610 cm^‐1^), an antiparallel β‐sheet (1,680 cm^‐1^), and a turn or hairpin (1692 cm^‐1^). PaH displayed notably reduced β‐sheets (1625, 1637, and 1694 cm^‐1^) and α‐helical (1652 cm^‐1^) structures, while the unordered coil at 1649 cm^‐1^ and the parallel β‐fold structure at 1682 cm^‐1^ became distinct peaks. Therefore, in addition to the β‐sheets, β‐folds were a major secondary structure in PaH. In the NH spectrum, the intensity of the bands at 1682 and 1694 cm^‐1^ decreased remarkably. The four MLP bands located near 1625, 1637, 1652, and 1667 cm^‐1^ shifted to 1627 (β‐strand), 1,640, 1656, and 1671 (α‐helices) cm^‐1^, respectively. Simultaneously, a new band at 1621 (intermolecular β‐sheet) appeared. TH looked quite similar to MLP, indicating that structural changes in MLP after trypsin hydrolysis were unremarkable. Overall, the secondary structures of MLP were changed after hydrolysis by different enzymes. Additionally, the structural changes differed by enzyme, possibly owing to enzyme specificity.

### 3.5 Amino acid composition analysis

As shown in Table 3, the amino acid composition of MLP changed after hydrolysis by different proteases. In addition to NH and TH, the TAA in other hydrolysates decreased significantly, especially FH and PaH. This is mainly because the enzymatic hydrolysis breaks down the peptide bonds in MP and hydrolyzes large proteins or peptides into smaller peptides and amino acids. During the dialysis process, some free amino acids could pass through the dialysis bag and be removed from the solution. As shown in Figure 2 and Table 2, after the enzymatic hydrolysis of MLP by Papain and Flavourzyme, the macromolecules were all hydrolyzed into small peptides (0.3–0.5 kda) and free amino acids, resulting in a large loss of amino acids and a significant reduction in TAA. This result is similar with that of Ao & Li, (Ao & Li, 2013) who reported that the amino acid content in residual peptides of casein (free amino acids were removed using gel filtration chromatography) exhibited significant reduction (*p* < .01). In these samples, Glu was the most abundant amino acid, followed by Asp (except for PaH). In terms of hydrophobic amino acids (HAAs), TH, NH, PrH, and AH exhibited relatively high amounts of Leu, Ala, Val, Ser and Phe, with significantly higher total HAA than PaH and FH (*p* < .05). A similar result was found for aromatic amino acids (AAAs). Among these hydrolysates, NH, AH, and PrH displayed a similar amino acid pattern. It was interesting to note that PaH exhibited higher Met and Ala, as well as lower Tyr and Cys compared with the other hydrolysates.

**TABLE 3:** Amino acid compositions of mulberry leaf protein hydrolysates

Amino acids	Amino acid contents (g kg^−1^of protein)						
	MLP	AH	PrH	NH	FH	PaH	TH
Asp	107.1 ± 6.5^a^	82.1 ± 3.8^c^	84.6 ± 5.6^c^	100.5 ± 1.23^b^	64.5 ± 1.6^d^	36.4 ± 1.3^e^	101.2 ± 2.5^ab^
Thr	58.9 ± 1.5^a^	42.1 ± 1.1^b^	43.1 ± 1.7^b^	49.9 ± 2.35^ab^	32.5 ± 1.4^c^	31.7 ± 3.1^c^	52.0 ± 2.2^ab^
Ser	55.0 ± 2.1^a^	37.7 ± 4.2 ^b^	39.0 ± 1.6^b^	45.9 ± 1.54^b^	29.6 ± 0.6^c^	36.4 ± 2.2^b^	48.6 ± 3.1^ab^
Glu	144.5 ± 6.7^a^	116.3 ± 7.2^c^	118.9 ± 4.2^c^	129.0 ± 3.53^b^	93.7 ± 2.5^d^	100.0 ± 4.6^d^	139.1 ± 7.1^ab^
Pro	50.2 ± 0.5^a^	40.3 ± 1.5^ab^	41.1 ± 1.0^ab^	46.7 ± 0.91^a^	30.4 ± 0.8^b^	28.9 ± 1.1^b^	48.2 ± 4.0^a^
Gly	63.4 ± 3.0^a^	50.6 ± 3.1^bc^	52.1 ± 2.6^bc^	54.1 ± 3.51^ab^	40.0 ± 1.9 cd	29.7 ± 1.5^d^	60.2 ± 2.2^a^
Ala	58.7 ± 2.3^a^	48.0 ± 1.2^c^	49.4 ± 3.6^bc^	50.2 ± 1.42^bc^	35.1 ± 1.3^d^	62.9 ± 4.3^a^	53.6 ± 4.2^ab^
Val	30.7 ± 1.3^a^	36.3 ± 1.6^a^	32.9 ± 1.7^a^	38.0 ± 4.01^a^	29.4 ± 1.6^a^	11.7 ± 0.5^b^	34.7 ± 2.2^a^
Met	10.8 ± 1.1 ^b^	15.9 ± 1.3^ab^	16.8 ± 1.1^ab^	16.5 ± 1.62^ab^	12.6 ± 1.2^b^	22.5 ± 1.6^a^	12.2 ± 0.6^b^
Ile	31.3 ± 2.5 ^a^	38.2 ± 2.3^a^	36.5 ± 1.6^a^	38.1 ± 1.98^a^	34.1 ± 2.1^a^	37.8 ± 1.8^a^	37.7 ± 3.6^a^
Leu	63.9 ± 4.0 ^a^	69.2 ± 5.6^a^	67.4 ± 4.2^a^	65.3 ± 3.65^a^	51.8 ± 3.1^b^	47.0 ± 1.7^b^	67.6 ± 5.3^a^
Tyr	42.2 ± 3.6 ^a^	42.6 ± 4.1^ab^	42.8 ± 2.6^ab^	42.9 ± 1.54^ab^	35.0 ± 1.8^b^	16.0 ± 1.0^c^	50.5 ± 4.6^a^
Phe	33.4 ± 1.0 ^ab^	38.4 ± 5.1^a^	40.6 ± 2.3^a^	34.5 ± 2.23^ab^	26.7 ± 1.3^b^	34.8 ± 2.6^ab^	34.4 ± 2.4^ab^
Arg	38.9 ± 1.7^a^	31.8 ± 1.6^ab^	35.8 ± 2.1^ab^	39.3 ± 3.22^a^	25.1 ± 1.8^b^	9.4 ± 1.1^c^	39.4 ± 2.6^a^
Lys	16.8 ± 1.4 ^a^	12.9 ± 1.2^a^	15.2 ± 1.7^a^	16.4 ± 1.22^a^	9.8 ± 0.6^a^	14.1 ± 1.6^a^	15.1 ± 1.3^a^
His	35.0 ± 2.5^a^	31.4 ± 1.9^a^	32.8 ± 2.3^a^	37.5 ± 2.57^a^	21.1 ± 3.1^b^	21.5 ± 1.3^b^	32.4 ± 2.5^a^
Cys	18.7 ± 1.8^a^	8.4 ± 0.6^b^	10.1 ± 1.3^b^	15.3 ± 1.51^a^	7.1 ± 0.7^b^	4.2 ± 0.5^c^	11.8 ± 1.6^b^
HAA^1^	339.9 ± 8.0^b^	337.3 ± 10.1^b^	339.6 ± 8.6^b^	347.5 ± 4.01^a^	262.2 ± 5.2^c^	265.8 ± 6.3^c^	350.7 ± 13.2^a^
AAA^2^	75.6 ± 3.5^a^	81.0 ± 4.2 ^a^	83.4 ± 2.8 ^a^	77.4 ± 4.22^a^	61.7 ± 1.4 ^b^	50.8 ± 2.2 ^c^	84.9 ± 5.6 ^a^
TAA^3^	859.5 ± 10.2^a^	742.2 ± 14.1^b^	759.1 ± 14.6^b^	820.1 ± 6.02^a^	578.5 ± 12.1^c^	545.0 ± 14.6^c^	838.7 ± 24.0^a^

Note

Data are expressed as mean ± *SD* (*n* = 3), different superscripts on the same line refer to the significant differences at *p* < .05. AH, PrH, PaH, FH, NH, TH: the hydrolysates of mulberry leaf protein hydrolyzed by alcalase, protamex, papain, flavourzyme, neutrase and trypsin, respectively. Different superscripts (a,b,c, d and e) on the same line refer to the significant differences at *p* < .05.

^1^ Hydrophobic amino acids (Ala, Val, Ile, Leu, Tyr, Phe, Pro, Met and Cys).

^2^ Aromatic amino acids (Phe and Tyr).

^3^ Total amino acids.

### 3.6 Antioxidant activity of MLP and MLPHs

**FIGURE 4:**
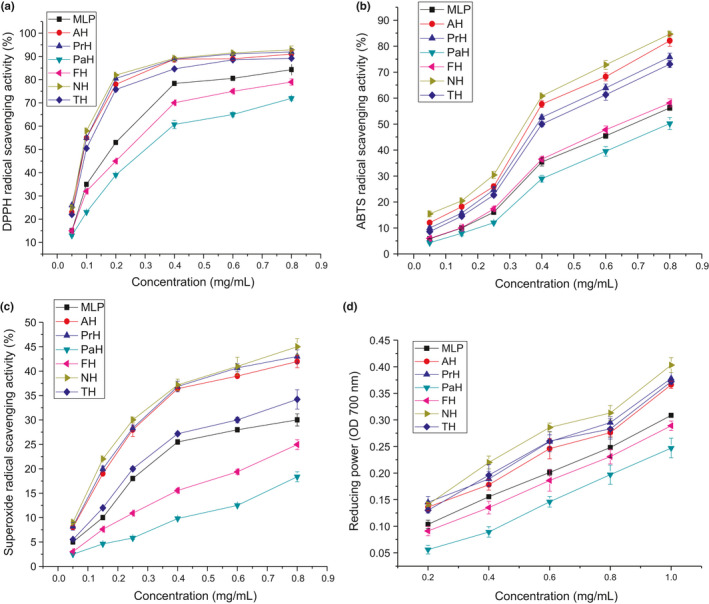
The DPPH (a), ABTS (b) and Superoxide (c) radical scavenging activity, and reducing power (d) of MLP and MLP hydrolysates at various concentrations. MLP: mulberry leaf protein; AH, PrH, PaH, FH, NH, and TH: the hydrolysates of MLP hydrolyzed by alcalase, protamex, papain, flavourzyme, neutrase, and trypsin, respectively. The data with different letters in the same test are significantly different (*p* < .05)

The RSA and reducing power of MLP and MLPHs were dose‐dependent and reached their maxima at the concentration of 0.8 mg/ml and 1.0 mg/ml, respectively (Figure 4). With the exception of PaH and FH, the RSA value of MLPHs was significantly higher than that of MLP. In the DPPH radical quenching analysis, the IC50 values of NH (0.087 mg/ml), PrH (0.091 mg/ml), AH (0.092 mg/ml), and TH (0.099 mg/ml) were significantly lower than those of FH (0.252 mg/ml) and PaH (0.307 mg/ml) (Table 2). DPPH radical quenching ability of protein hydrolysates is associated with the balance between high levels of hydrophobicity and good diffusivity of radicals in reaction medium. (Bamdad et al., 2011) After papain and Flavourzyme hydrolysis, the large fragments and polypeptides of MLP were digested into hydrophilic oligopeptides and amino acids (Table 2), and the increased polarity may make it more difficult to capture DPPH radicals. (You et al., 2010) The ABTS radical scavenging ability of MLP and MLPHs was in the following order: NH > AH > PrH > TH > MLP > FH > PaH (Figure 4b). Similar trends were observed for superoxide radical scavenging activity. NH, PrH, and AH had the highest O_2_.^‐^scavenging abilities, followed by TH, MLP, FH, and PaH (Figure 4c). The radical scavenging abilities of the MLPHs were significantly correlated with the YSP (*p* < .01, *r* ranged from 0.956 to 0.985, Table S1), as the higher the YSP, the higher the radical quenching ability. Therefore, optimal DH and YSP could improve the radical capturing ability of MLP. Similar results were observed by You et al. (You et al., 2009) In addition, AAA have been used as hydrogen donors to quench free radicals. HAA, especially His, Tyr, Trp, and Pro, are the most important residues in radical scavenging ability. (Wang et al., 2007) Table 3 showed that the HAA and AAA contents in PaH and FH were significantly lower than that in MLP (*p* < .05). The decrease in the level of HAA and AAA may decrease the level of antioxidant activity.

Similar to the RSA results, the reducing capacities of NH, AH, PrH, and TH were higher than that of MLP (Figure 4d). At the concentration of 1.0 mg/ml, the reducing value of NH was the highest (0.403), followed by PrH (0.378), TH (0.372), and AH (0.366), and PaH was the lowest (0.267). These values were superior to that of pea peptides (0.012) (Pownall et al., 2010) and barley hordein hydrolysates (0.137) (Bamdad et al., 2011) at 1 mg/ml. It is worth noting that both the radical scavenging ability and reducing power of PaH and FH were lower than that of MLP. This suggests that papain and Flavourzyme hydrolysis of MLP not only failed to improve antioxidant activity, they actually decreased it. In addition, PaH and FH had remarkably higher DH values but significantly lower antioxidant activities than TH. This indicated that higher DH did not guarantee a higher antioxidant activity. A similar conclusion has been reported by Barkia et al. (Barkia et al., 2010).

In addition, although MLPH is mainly composed of soluble peptides that play a major role in antioxidant activity, non‐peptide components in MLPH may affect the antioxidant activity. As shown in Table S1, DPPH radical scavenging ability and reducing ability were significantly correlated with TPC, with r values of 0.929 and 0.865, respectively. As for how to affect, it needs to be further studied.

### 3.7 Effects of hydrolysis time, substrate concentration and enzyme to substrate ratio on the antioxidant activity of hydrolysates

**FIGURE 5:**
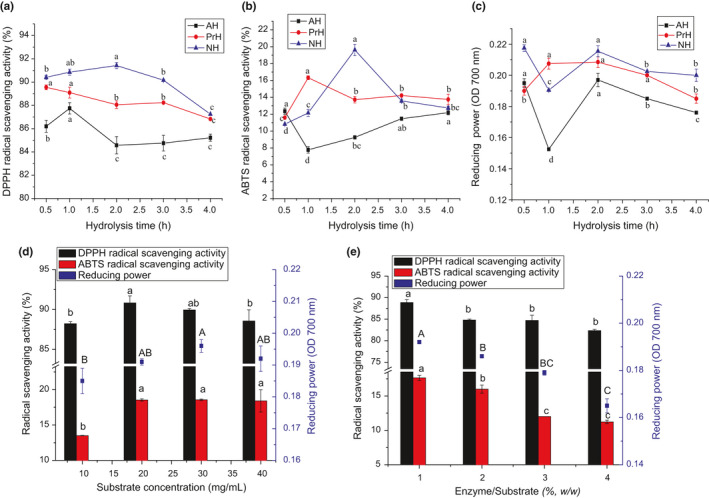
Effects of hydrolysis time, enzyme/substrate, and substrate concentration on the antioxidant activity of mulberry leaf protein hydrolysates. a–c: the DPPH (a), ABTS radical quenching activity (b) and reducing power (c) of AH, NH, and PrH at different hydrolysis time (0.3 mg/ml). d–e: the antioxidant activity of NH at different substrate concentrations (d) and enzyme to substrate ratios (e). AH, PrH, and NH: the hydrolysates of MLP hydrolyzed by Alcalase, Protamex and Neutrase, respectively. The data with different letters in the same test are significantly different (*p* < .05)

Based on the results of DH, YSP, and antioxidant activities of the six MLPHs, it was determined that protamex, alcalase, and neutrase were the most efficient proteases to hydrolyze MLP for the production of antioxidant peptides. To investigate the effects of duration of hydrolysis on the antioxidant activity of AH, PrH, and NH, the DPPH and ABTS radical quenching abilities and reducing power of these three hydrolysates were tested after various durations of hydrolysis. In the DPPH radical scavenging assay (Figure 5a), NH showed higher activity than AH and PrH, which peaked at 2 hr (92.42%). The DPPH radical scavenging ability of AH at 1 hr was significantly stronger than at other times (*p* < .05). In the ABTS radical quenching assay (Figure 5b), AH, PrH, and NH reached their highest values at 0.5 hr (12.36%), 1 hr (16.32%), and 2 hr (19.61%), respectively. With regard to the reducing ability, NH and AH presented similar change trends, which decreased abruptly during the first 1 hr of hydrolysis and increased during the next hour (Figure 5c). This pattern is similar with that reported for Flavourzyme‐hydrolyzed barley hordein. (Bamdad et al., 2011) In contrast to AH and NH, PrH displayed negligible changes in reducing capacity within 3 hr. Combined with the DPPH and ABTS radical scavenging results, it is clear that the antioxidant activities of these samples were not improved but reduced by further hydrolysis, with AH, PrH, and NH displaying their best antioxidant activity at 0.5, 1, and 2 hr, respectively. Therefore, consistent with the conclusion of You et al., (You et al., 2009) limited hydrolysis of MLP can result in better antioxidant abilities than extensive hydrolysis. The antioxidant activity of NH at 2 hr was significantly higher than that of AH at 0.5 hr and PH at 1.0 hr, so NH was selected for further optimization of enzymatic hydrolysis. As shown in Figure 5d and e, the antioxidant activity of NH was the highest when the substrate concentration was 20 mg/ml and the enzyme to substrate ratio was 1%.

## 4 CONCLUSIONS

Mulberry leaf protein hydrolysates prepared with neutrase, alcalase, and protamex were preferable to other enzymes, and showed significantly higher antioxidant activity than MLP. The hydrolysates of these three enzymes exhibited similar amino acid compositions and Mw distributions. There was no clear relationship between Mw and DH, and higher DH did not guarantee higher YSP or higher antioxidant activity. Furthermore, limited hydrolysis of MLP resulted in better antioxidant ability than extensive hydrolysis. The results of optimization of enzymatic hydrolysis conditions showed that the antioxidant activity of NH was the best when the substrate concentration was 20 mg/ml, the ratio of enzyme to substrate was 1%, and the enzymatic hydrolysis time was 2 hr. Further research, including the isolation and purification of antioxidant peptides from NH, will be necessary; however, these results suggest that NH may be a promising antioxidant in food products.

## CONFLICT OF INTEREST

None.

## ETHICAL APPROVAL

This article does not contain any studies with human or animal subjects.

## Supporting information

Supplementary Material

## Data Availability

The data that support the findings of this study are available from the corresponding author upon reasonable request.
