# Supplementary material for: Effects of enzymatic hydrolysis on physicochemical property and antioxidant activity of mulberry (Morus atropurpurea Roxb.) leaf protein
Source: Food Sci Nutr. 2021 Aug 25;9(10):5379–90. doi: 10.1002/fsn3.2474 (PMC8498073; doi:10.1002/fsn3.2474)
Supplement: Supplementary file 1 — Supplementary Material [file FSN3-9-5379-s001.docx]

**Table S1 The correlation analysis of various physicochemical indexes and antioxidant indexes**

| Items | YSP^1^ | Radical scavenging activity | | | Reducing power | Protein content | Protein yield | DH^2^ | Total  sugar | TPC^3^ |
| --- | --- | --- | --- | --- | --- | --- | --- | --- | --- | --- |
|  |  | DPPH^.^ | O_2_^.-^ | ABTS^+.^ |  |  |  |  |  |  |
| YSP | 1 | 0.916** | 0.985** | 0.956** | 0.81 | 0.572 | 0.59 | 0.528 | 0.127 | 0.81 |
| DPPH**^.^** |  | 1 | 0.944^**^ | 0.923^**^ | 0.965^**^ | 0.300 | 0.325 | 0.214 | 0.375 | 0.929** |
| O**_2_^.-^** |  |  | 1 | 0.957^**^ | 0.864^*^ | 0.578 | 0.599 | 0.522 | 0.147 | 0.811^*^ |
| ABTS**^+.^** |  |  |  | 1 | 0.882^*^ | 0.552 | 0.566 | 0.434 | 0.104 | 0.759 |
| Reducing |  |  |  |  | 1 | 0.201 | 0.227 | 0.089 | 0.447 | 0.865^*^ |
| Protein content |  |  |  |  |  | 1 | 0.999^**^ | 0.939^**^ | -0.649 | 0.01 |
| Protein yield |  |  |  |  |  |  | 1 | 0.943^**^ | -0.622 | 0.034 |
| DH |  |  |  |  |  |  |  | 1 | -0.512 | -0.015 |
| Total sugar |  |  |  |  |  |  |  |  | 1 | 0.584 |
| TPC |  |  |  |  |  |  |  |  |  | 1 |

YSP^1^: yield of soluble peptides；DH：the maximum degree of hydrolysis at 4 h；TPC: total phenolic content, grams of gallic acid equivalents (GAE) per 100 grams of dry material (g GAE/100g);

** significant correlation at the level of 0.01 (two-tail);* significant correlation (two-tail) at the level of 0.05.
